# Seroprevalence, Risk Factors, and Environmental Correlates of *Babesia caballi*, *Toxoplasma gondii*, and *Coxiella burnetii* in Equids from Southwestern Greece

**DOI:** 10.3390/pathogens15070703

**Published:** 2026-07-03

**Authors:** Antonia Touloudi, Alexios Giannakopoulos, Panagiota Tyrnenopoulou, Athanasios Siasios, Zoi Athanasakopoulou, Garyfallenia Tsinopoulou, Marina Sofia, Vassiliki Spyrou, George C. Fthenakis, Charalambos Billinis, Dimitrios C. Chatzopoulos

**Affiliations:** 1 Department of Public and One Health, University of Thessaly, 43100 Karditsa, Greece; atoul@uth.gr (A.T.); gtsinopoulou@uth.gr (G.T.); 2 Faculty of Veterinary Science, University of Thessaly, 43100 Karditsa, Greece; algiannak@uth.gr (A.G.); ptyrnenop@uth.gr (P.T.); zathanas@uth.gr (Z.A.); msofia@uth.gr (M.S.); gcf@uth.gr (G.C.F.); billinis@uth.gr (C.B.); 3 Independent Researcher, 50100 Kozani, Greece; thanasissiasios@hotmail.com; 4 Faculty of Animal Science, University of Thessaly, 41110 Larissa, Greece; vasilikispyrou@uth.gr

**Keywords:** equids, ecological niche modelling, seroprevalence, *Babesia caballi*, *Coxiella burnetii*, *Toxoplasma gondii*

## Abstract

Equids, primarily horses, are mostly used for recreational purposes, although in some rural areas they also serve as working animals, maintaining close and frequent contact with humans. Their risk of exposure to vector-borne and zoonotic pathogens can be affected by host-related factors, management practices and environmental conditions. This study aimed to investigate the seroprevalence and associated risk factors for infections by *Babesia caballi*, *Toxoplasma gondii*, *Coxiella burnetii*, and *Borrelia burgdorferi* sensu lato in equids from Southwestern Greece. A total of 159 equids were tested using commercial serological assays. Weighted prevalence estimates were applied to account for unequal sampling. Associations were assessed using chi-square tests and logistic regression. Ecological niche modelling was employed to evaluate geographic patterns and environmental correlates. Seroprevalence was highest for *B. caballi* (8.81%), followed by *T. gondii* (7.55%) and *C. burnetii* (1.26%). No seropositive animals were detected for *B. burgdorferi* sensu lato. Ecological niche modelling showed acceptable predictive performance for *B. caballi*, with BIO14 and BIO6 emerging as the main environmental predictors. In contrast, the *T. gondii* model exhibited unacceptable predictive performance, and its environmental associations should therefore be interpreted cautiously. Complementary Random Forest analyses yielded comparable environmental rankings but showed higher classification performance for *T. gondii* than for *B. caballi*. Overall, the findings contribute to understanding pathogen exposure patterns in equids and underscore the importance of integrating epidemiological and environmental data in surveillance efforts.

## 1. Introduction

Monitoring infections in animal populations helps assessing pathogen circulation, transmission dynamics, and spillover risk [1]. To date, several animal species have been found to act as sentinels, providing early warning of emerging public health threats. For example, systematic surveillance of wild birds has played a key role in the early detection of avian influenza and flavivirus outbreaks (including West Nile virus (WNV) and Usutu virus) across Europe and North America [2,3,4], while rodents and small mammals have been instrumental in tracking the spread of hantaviruses and other zoonotic agents [5]. Through their serological and microbiological profiles, these sentinel species can indicate whether a pathogen is established within an ecosystem or whether its emergence is associated with environmental changes and anthropogenic pressures. Furthermore, the study of infection dynamics in animal hosts provides valuable information on the effectiveness of biosecurity measures and control strategies, including vector control, vaccination, and restrictions on animal movement [6].

Equids have long played an important role in human societies. This role varies across regions and socioeconomic contexts. In developing countries, horses, donkeys, and mules remain essential for agriculture, livestock management, and transport. In contrast, in more developed regions they are linked to leisure, sport, and, increasingly, to therapeutic applications such as equine-assisted interventions [7,8,9]. In many countries, including Greece, equids hold a strong cultural and social presence, often tied to local heritage and community identity [10].

In this context, equids may provide useful information regarding environmental circulation of pathogens and vector exposure within shared ecosystems. For instance, these species have been implicated in the epidemiology of several arboviruses, serving as amplification or incidental hosts in outbreaks across Europe and North America [11]. Their exposure to ticks and biting flies further positions them within the transmission cycles of haemoparasites and intracellular bacteria of veterinary and public health importance [12,13]. In this sense, equids can serve as hosts, as well as sentinels reflecting environmental pathogen circulation and vector activity.

The present study focuses on four pathogens of veterinary and public health relevance: *Babesia caballi*, *Coxiella burnetii*, *Toxoplasma gondii*, and *Borrelia burgdorferi* sensu lato. *B. caballi* is a tick-borne haemoparasite infecting equine erythrocytes, with distribution closely linked to the ecology of its vectors and endemic presence in southern Europe and the Mediterranean basin [14,15,16,17]. Due to its impact on international horse movement and trade, equine piroplasmosis has been classified as a notifiable disease by the World Organization for Animal Health [18]. *B. caballi* is considered primarily a veterinary pathogen and current evidence does not support its role as a confirmed zoonotic agent [19]. In contrast, *T. gondii* is a globally distributed zoonotic protozoan pathogen capable of infecting nearly all warm-blooded animals, with environmental contamination driven by oocyst shedding from felids [20]. Clinical toxoplasmosis in horses is considered rare, mainly resulting in neurologic disorders in immunosuppressed or debilitated animals. In addition, documented cases of *T. gondii* infection following consumption of contaminated equine meat have raised public health concerns, especially where meat inspection and cooking practices are insufficient [21]. *C. burnetii*, the causative agent of Q fever, is a highly contagious pathogen transmitted mainly via aerosolized particles from infected animals and has also been detected in equids as incidental hosts [22]. Clinical signs in horses are rare and non-specific, though fever, abortion, and lethargy have been reported. Finally, *B. burgdorferi* sensu lato, the aetiological agent of Lyme borreliosis, is a particularly important tick-borne zoonosis in the Northern Hemisphere, with equids acting as indicators of pathogen presence in shared environments [23].

The aim of the study was to investigate the seroprevalence and associated risk factors of these pathogens in equids from southwestern Greece, a region of particular epidemiological interest due to its geographic position as a transition zone between Europe and North Africa, its diverse ecological characteristics, and its history of vector-borne disease outbreaks. Furthermore, ecological niche modelling and environmental analyses were conducted to investigate potential environmental correlates of pathogen distribution, alongside the evaluation of management-related risk factors associated with pathogen exposure.

## 2. Materials and Methods

### 2.1. Study Area, Sample Collection, and Serological Testing

Between August and November 2023, a total of 159 equids (152 horses, 4 donkeys, and 3 ponies) were sampled from 55 stables across the regions of Peloponnese and West Greece. Sampling was conducted following relevant requests from animal owners, who contacted veterinarians as part of routine screening for antibodies against *West Nile Virus* infection. Two veterinarians visited each participating stable and performed a standardized clinical examination and blood collection procedure. Only animals considered clinically healthy at the time of sampling were included in the study and sampled. The geographic coordinates of each sampling site were recorded to support subsequent environmental analyses.

In parallel with sample collection, horse owners completed a structured questionnaire covering animal data, details regarding its use (e.g., leisure, working), implementation of ectoparasite control measures, and medical history. These data were used to explore potential associations between serological findings and management or environmental risk factors.

Blood samples were collected aseptically from the jugular vein and transported to the laboratory within 24 h under controlled conditions. Upon arrival, samples were centrifuged for serum separation, aliquoted, and stored at −20 °C until testing.

Serological testing was performed using commercially available ELISA kits to detect antibodies against *B. caballi*, *T. gondii*, *C. burnetii* and *B. burgdorferi* sensu lato. The assays used were, respectively, the cELISA *B. caballi* Antibody Test Kit (VMRD Inc., Pullman, WA, USA (for equid samples: sensitivity 100%, specificity: 100% [24])), the ID Screen^®^ Toxoplasmosis Indirect Multi-Species (IDvet, Grabels, France (for equid samples: sensitivity 76.5%, specificity: 87.7% [25])), the ID Screen^®^ Q Fever Indirect (IDvet, Grabels, France (for all animal samples: sensitivity > 94%, specificity: 100% [26], as used in horses [27])) and the ID Screen^®^ Lyme Disease (Borreliosis) Indirect (IDvet, Grabels, France (for equid samples: sensitivity 96.0%, specificity: 100% [28])). All assays were commercially available diagnostic tests that had been validated by the respective manufacturers for their intended use. Information regarding reported diagnostic sensitivity and specificity is available in the respective manufacturer documentation.

Samples yielding doubtful results were retested. Samples that remained inconclusive after repeat testing were classified as seronegative according to the manufacturer’s interpretation criteria. Overall, such results were observed in 2 of the 159 samples tested with the ID Screen^®^ Toxoplasmosis Indirect Multi-Species assay for the detection of antibodies against *T. gondii* (1.26%).

### 2.2. Statistical Analysis

All statistical analyses were conducted using IBM SPSS Statistics version 29 (IBM Corp., Armonk, NY, USA). Seroprevalence for each pathogen was expressed as a percentage at both the individual animal and stable level, with corresponding 95% confidence intervals (CIs). A stable was classified as positive when at least one equid therein tested seropositive. Further, co-exposure between pathogens was explored using odds ratios calculated from 2 × 2 contingency tables, assessing potential associations between seropositivity to different pathogens.

To account for unequal sampling across stables, a weighted prevalence approach was applied. Specifically, within-stable seroprevalence was multiplied by the total number of equids housed in each stable, and the resulting weighted counts were summed to estimate the overall infection burden. The adjusted prevalence was then calculated by dividing this value by the total equine population across all stables (*n* = 292), allowing for more representative population-level estimates.

In the analysis of association between breed and seropositivity, only breeds with at least four individuals were considered. In this context, ponies and donkeys were excluded.

Associations between pathogen seropositivity and categorical variables, including sex, breed, age category, housing type, presence of farm animals, companion animals (cats and dogs), wildlife exposure, horse use, ectoparasiticide treatment, and prior clinical history, were assessed using Pearson’s chi-square tests. The presence of farm animals (sheep and goats), companion animals (cats and dogs), and wildlife within the stable environment was classified as a binary variable (present/absent). Ectoparasiticide treatment and prior clinical history were also classified as binary variables (yes/no). Equid use was classified as either leisure or working animal. Housing conditions were classified into three categories reflecting increasing levels of environmental exposure: (1) fully enclosed stables, (2) stables with partial outdoor access, and (3) permanent outdoor housing without formal shelter. Age was considered both as a continuous variable and as a categorical variable; for the latter assessment, animals were classified into four biologically relevant classes: young (<4 years), young adult (4–10 years), adult (11–14 years), and senior (>14 years). Differences in age distribution between seropositive and seronegative animals were examined using the Mann–Whitney U test, whereas chi-square tests were used for comparisons across age categories.

For stable-level analyses, associations between pathogen seropositivity and housing type, presence of farm animals, companion animals (cats and dogs), wildlife, and routine ectoparasiticide treatment were initially assessed using Pearson’s chi-square tests. Housing type was classified according to the three housing categories described previously, whereas the presence of farm animals, companion animals, wildlife, and routine ectoparasiticide treatment was recorded as binary variables (present/absent or yes/no, as appropriate). Subsequently, separate univariable binary logistic regression models were constructed using stable infection status (positive/negative) as the dependent variable and housing type or presence of farm animals as independent variables. These two variables were selected based on their potential biological relevance to pathogen exposure, as outdoor access and mixed-animal environments may increase exposure to vectors and environmental contamination sources. Multivariable analysis was not carried out because of the small rate of seropositive samples (<9% of samples) for all outcomes evaluated. All statistical tests were two-tailed, with significance set at *p* < 0.05.

### 2.3. Environmental Analysis

Environmental analyses were performed for *B. caballi* and *T. gondii*. To investigate environmental determinants potentially associated with pathogen occurrence, a comprehensive ecological modelling framework was developed, including bioclimatic variables, elevation and CORINE land-cover category. Climatic information consisted of the 19 standard bioclimatic variables (BIO1–BIO19) obtained from the WorldClim version 2.1 database at 2.5 arc-minute spatial resolution (~5 km) [29,30]. Elevation data were obtained from the WorldClim digital elevation model at the same spatial resolution as the bioclimatic layers. Land-use information was derived from the CORINE Land Cover 2018 database [31], and all land-cover classes present within the study area were retained during the initial environmental extraction process (Table 1). In order to minimize redundancy among predictors and reduce the risk of model overparameterization, all environmental variables were evaluated simultaneously using an iterative Variance Inflation Factor (VIF) analysis. Variables exhibiting excessive multicollinearity were removed until all retained predictors satisfied a conservative VIF threshold (<10).

Ecological niche modelling was performed using MaxEnt version 3.4.4, applying default regularization settings, random seed initialization, and cross-validation procedures (10-fold for *B. caballi* and 9-fold for *T. gondii*). MaxEnt models were developed using presence-only occurrence records and background environmental points sampled from the study area. To complement the presence-only modelling approach, Random Forest classifiers were implemented in Python 3.12.9. using the scikit-learn library (1000 trees, balanced class weights, stratified 5-fold cross-validation, random state = 42) using observed presence–absence data derived from serological results. Jackknife analyses were conducted within the MaxEnt framework to assess the relative contribution and unique explanatory value of individual environmental predictors. Model performance for both approaches was assessed using the area under the receiver operating characteristic curve (AUC).

## 3. Results

### 3.1. Seroprevalence and Descriptive Findings

**Figure 1:**
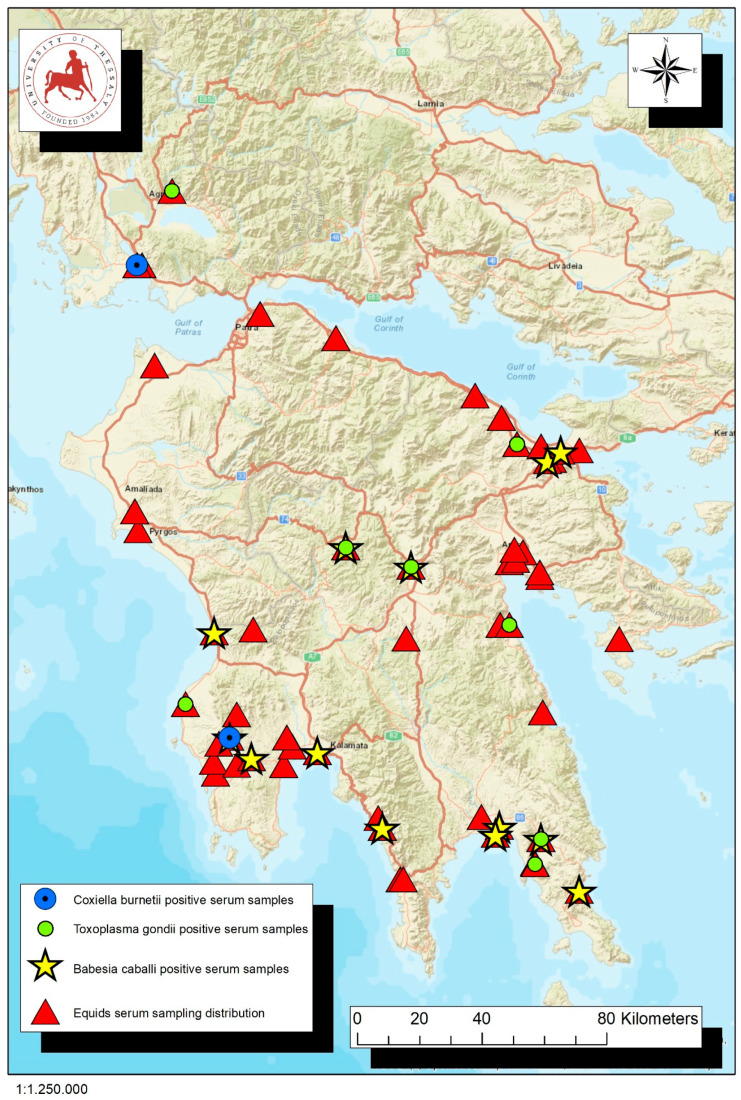
Spatial distribution of equid sampling sites and seropositive animals.

The serological findings revealed notable variation in pathogen exposure among the study population. To provide spatial context, the distribution of sampling locations and seropositive animals is presented in Figure 1.

At individual animal level, *B. caballi* was the most frequently detected pathogen, with a seroprevalence of 8.81% (14/159; 95% CI: 5.32–14.24%), followed by *T. gondii* at 7.55% (12/159; 95% CI: 4.37–12.73%) and *C. burnetii* at 1.26% (2/159; 95% CI: 0.35–4.47%). No equids tested seropositive for *Borrelia burgdorferi* sensu lato (0/159; 95% CI: 0.00–2.36%). Weighted prevalence estimates resulted in slightly higher values, reaching 9.54% for *B. caballi*, 7.20% for *T. gondii*, and 1.10% for *C. burnetii*.

At stable level, *B. caballi* was also found to be the most widespread pathogen, with a prevalence of 23.63% (13/55; 95% CI: 14.37–36.35%), followed by *T. gondii* at 18.18% (10/55; 95% CI: 10.19–30.33%) and *C. burnetii* at 3.63% (2/55; 95% CI: 1.00–12.32%).

Co-seropositivity with *B. caballi* and *T. gondii* was identified in four equids from four different stables, corresponding to a co-exposure rate of 2.52%. Odds ratio analysis indicated that horses seropositive for *B. caballi* had greater odds of being seropositive for *T. gondii* compared with seronegative animals (OR = 7.28, 95% CI: 1.84–28.85, *p* = 0.011), suggesting a positive association between exposure to the two pathogens.

### 3.2. Factors Associated with Seropositivity

#### 3.2.1. Animal-Related Factors

Sex-associated differences were observed for *B. caballi*, with a significantly higher seroprevalence in intact males (17.46%) compared to females (2.89%) and castrated males (3.70%) (*χ*^2^ = 9.75, *p* = 0.008). In contrast, no significant association between sex and *T. gondii* seropositivity was found (*χ*^2^ = 0.02, *p* = 0.99). Breed-based analysis, restricted to horse populations with adequate representation (*n* = 147), revealed no statistically significant differences in seroprevalence for both *B. caballi* and *T. gondii* (*p* = 0.97 and *p* = 0.95, respectively). Moreover, age was not significantly associated with seropositivity for both pathogens when evaluated as a continuous variable (Mann–Whitney U test: *B. caballi*, *p* = 0.21; *T. gondii*, *p* = 0.30) or as a categorical variable (*χ*^2^: *B. caballi*, *p* = 0.46; *T. gondii*, *p* = 0.66). Finally, also for both pathogens, the number of equids per stable was not significantly associated with seropositivity risk in logistic regression models (*B. caballi*, *p* = 0.36; *T. gondii*, *p* = 0.76) (Table 2).

#### 3.2.2. Management and Environmental Exposure Factors

At the animal level, housing conditions were the only management factor significantly associated with pathogen exposure, being linked to *T. gondii* seropositivity (*χ*^2^ = 6.53, *p* = 0.04). Equids maintained under semi-open housing conditions exhibited the highest seroprevalence. In contrast, no significant association was identified between housing conditions and *B. caballi* seropositivity.

The presence of farm animals was not significantly associated with seropositivity for either pathogen *(B. caballi*: *p* = 0.15; *T. gondii*: *p* = 0.09). Further, no significant associations were observed between pathogen seropositivity and any of the remaining variables examined, including exposure to companion animals or wildlife, horse use and routine ectoparasiticide treatment.

A comparable pattern was observed at the stable level. Housing conditions remained significantly associated with *T. gondii* seropositivity (*χ*^2^ = 6.92, *p* = 0.03), whereas no significant association was detected for *B. caballi.* Likewise, the presence of farm animals was not associated with seropositivity for either pathogen. Furthermore, neither the presence of companion animals, wildlife, equid use nor routine ectoparasiticide treatment showed a significant association with pathogen seropositivity (Table 3).

The stable-level logistic regression analyses largely corroborated the findings of the univariable analyses. Specifically, semi-open housing conditions were associated with significantly increased odds of *T. gondii* seropositivity compared with closed housing systems (OR = 7.50; 95% CI: 1.14–49.26; *p* = 0.03). Although a similar trend was observed for *B. caballi*, the association did not reach statistical significance (OR = 5.14; 95% CI: 0.76–34.69; *p* = 0.09). Also, the presence of farm animals was associated with increased odds of seropositivity for both pathogens (*B. caballi*: OR = 3.38; 95% CI: 0.66–17.28; *p* = 0.14; *T. gondii*: OR = 5.46; 95% CI: 0.64–47.01; *p* = 0.12), although neither association was statistically significant.

### 3.3. Ecological Analysis

#### 3.3.1. Model Performance

Following iterative VIF-based filtering, four variables satisfied the predefined multicollinearity threshold (<10) and were retained for subsequent analyses for both *B. caballi* and *T. gondii*. The final predictor set consisted of minimum temperature of the coldest month (BIO6; VIF = 4.75), precipitation of the driest month (BIO14; VIF = 9.91), land-cover category (VIF = 5.87) and elevation (VIF = 4.90). These variables were subsequently incorporated into both the MaxEnt ecological niche models and the Random Forest classification analyses.

The ecological niche modelling produced contrasting results for the two pathogens. For *B. caballi*, the replicated MaxEnt model showed moderate predictive performance, with an average test AUC of 0.705 (SD = 0.292) across cross-validation replicates. Variable contribution analysis identified BIO14 (precipitation of the driest month) as the dominant predictor, accounting for 64.3% of the average model contribution and 75.8% of permutation importance. Further, BIO6 (minimum temperature of the coldest month) was the second most influential predictor, contributing 35.7% to the model and 24.2% of permutation importance. Land-cover category and elevation showed no measurable contribution in the replicated MaxEnt model (Table 4).

Regarding *T. gondii*, the replicated MaxEnt model demonstrated unacceptable predictive performance, with a mean test AUC of 0.397 (SD = 0.233) across cross-validation replicates.

Random Forest analyses produced different predictive performances for the two pathogens. For *B. caballi*, the model showed limited predictive ability, with a mean AUC of 0.504 ± 0.141 across five cross-validation folds. Variable-importance analysis identified elevation as the most influential predictor (0.405), followed by BIO6 (0.264), land-cover category (0.169), and BIO14 (0.162). For *T. gondii*, the Random Forest model achieved an AUC of 0.730, whereas the corresponding MaxEnt model showed poor predictive performance. Elevation was again identified as the most influential predictor (0.435), followed by BIO6 (0.304), BIO14 (0.145), and land-cover category (0.117).

#### 3.3.2. Jackknife Analysis

**Figure 2:**
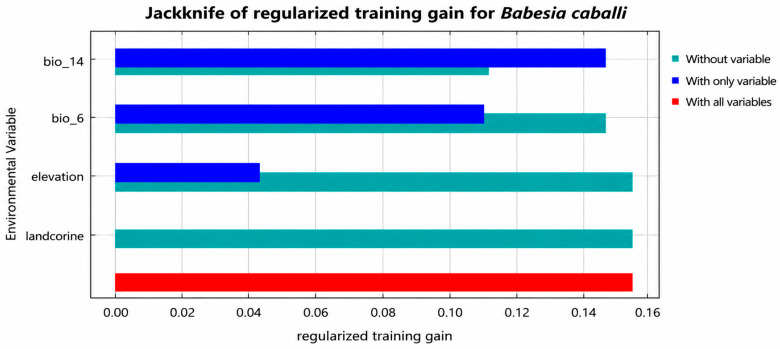
Jackknife test of variable importance for the ecological niche models of seropositivity against *B. caballi*. Bars represent the regularized training gain when each environmental variable is used in isolation and when it is omitted from the model.

**Figure 3:**
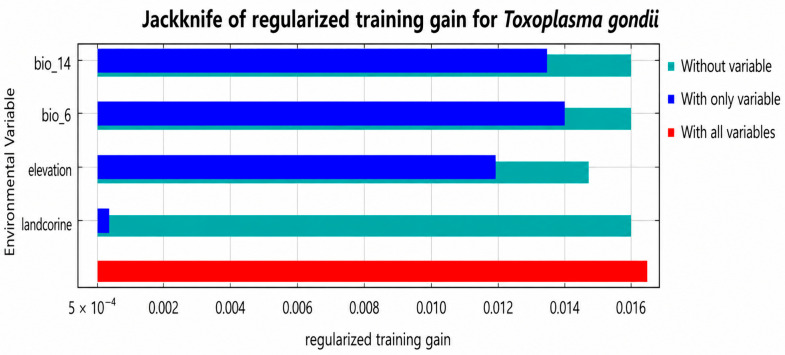
Jackknife test of variable importance for the ecological niche models of seropositivity against *T. gondii*. Bars represent the regularized training gain when each environmental variable is used in isolation and when it is omitted from the model.

Τhe Jackknife analyses were generally consistent with the variable-contribution results. For *B. caballi*, precipitation of the driest month (BIO14) provided the highest gain, when used in isolation and resulted in the greatest reduction in model gain when omitted, indicating that this variable contained the most unique predictive information (Figure 2). For *T. gondii*, BIO14 produced the highest gain when used alone, followed closely by BIO6, whereas elevation caused the largest decrease in gain when omitted (Figure 3).

## 4. Discussion

### 4.1. Prevalence of Seropositivity

The present study provides information on exposure of horses in Southwestern Greece to selected vector-borne and zoonotic pathogens. Antibodies against *B. caballi*, *T. gondii*, and *C. burnetii* were detected during the study, whereas no serological evidence of exposure to *B. burgdorferi* sensu lato was identified.

Despite the widely recognized significance of equine piroplasmosis, data on the circulation of *B. caballi* and other equine piroplasm species among equids in Greece have remained of limited extent. A study performed some 15 to 20 years ago [32] reported a *B. caballi* seroprevalence of 2.2% in mainland Greece, which is substantially lower than the seroprevalence observed in the present study. This discrepancy may be attributed to differences in geographic sampling, but also reflects ongoing ecological changes, particularly those related to climate shifts and the expanding distribution of tick vectors, which may have influenced pathogen transmission over time. In comparison with similar studies conducted in other Mediterranean countries, the observed seroprevalence of *B. caballi* was comparable to that reported in Italy (10.3%), but lower than the higher figures reported in Spain (20–50%), where favourable climatic conditions support the persistence and activity of tick vectors [33,34,35]. In contrast, data from Central European countries indicate that prevalence of equine piroplasmosis is generally lower, as highlighted by large-scale molecular and serological investigations conducted across Europe [36]. These differences can be largely attributed to reduced tick density, shorter vector seasons, and the implementation of more effective preventive management systems.

*T. gondii* seroprevalence in equids in our dataset is consistent with findings from the same region reported previously [32], where a prevalence of 6.25% was identified. Studies in other Mediterranean countries, have shown higher rates, with Italy (17.7–22.6%) and Spain (16.6%) reporting higher levels, while similar levels have been documented in Portugal (6.5%) [37,38,39]. In non-Mediterranean European countries, seroprevalence appears to vary considerably, ranging from very low levels in Sweden [40] to relatively higher levels reported in the Czech Republic [41]. In contrast, studies from Turkey [42] and North African countries [43,44] consistently have indicated higher seroprevalence rates, suggesting increased environmental exposure and transmission pressure in these regions.

Although horse meat consumption is prohibited in Greece, the practice remains common in some countries, and the presence of *T. gondii* in equids may represent a potential public health concern in countries where horse meat is consumed. Infected horses may harbor viable tissue cysts that can be transmitted to humans through consumption of raw or undercooked meat. This risk is supported by findings from Italy, where a seroprevalence of 17.6% was reported in slaughtered horses and parasite DNA was detected in edible tissues, indicating the potential for foodborne transmission [45]. Furthermore, the risk may be exacerbated by the illicit incorporation of horse meat into processed products, as highlighted during the 2013 European adulteration incident [46].

The seroprevalence of *C. burnetii* in equids in our study was 1.26%, consistent with reports from Slovakia (2.17%) [27], but lower than rates reported in Mediterranean countries, including France (4–12%) and Italy (15.8%) [15,47]. Globally, relevant prevalence varies widely, reaching 9.9% in Algeria, particularly in areas with close contact with small ruminants [48]. This is the first study assessing *C. burnetii* exposure in equids in Greece, providing information for future surveillance efforts. Notably, the two seropositive animals were used for leisure activities and had no direct contact with livestock, suggesting that indirect environmental exposure may be possible [49,50]. However, as only two seropositive animals were identified, no reliable conclusions can be drawn regarding associated ecological or epidemiological patterns of the pathogen.

In the present study, no seropositive horses for *B. burgdorferi* sensu lato were detected. This finding contrasts with previous reports from Greece and other European countries, where variable seroprevalence has been documented [51]. These discrepancies may reflect differences in geographic distribution, tick exposure, diagnostic methods, and sampling strategies.

Among the 159 equids tested, four animals were found to be seropositive against both *B. caballi* and *T. gondii*. Notably, these horses were housed in different stables, suggesting that co-exposure was not due to a localized outbreak, but, rather, reflects broader environmental or management-related factors. Interestingly, previous studies have reported the detection of *T. gondii* DNA in ticks, including *Ixodes* spp. [52]. However, the epidemiological significance of that finding remains controversial, and the present study does not provide evidence directly supportive of tick-mediated transmission.

Differences between individual- and stable-level prevalence can provide insights into the transmission dynamics of the pathogens. The higher stable-level prevalence for *B. caballi* suggests clustering of infection across holdings, consistent with localized, vector-driven transmission in environments that support tick persistence. In contrast, the less pronounced increase observed for *T. gondii* indicates a more diffuse pattern of exposure, mainly associated with widespread environmental contamination rather than within-stable transmission. This hypothesis is supported by the observed association between housing type and seropositivity at both the individual and stable levels, highlighting environmental access as a key risk factor.

### 4.2. Seropositivity Correlates

Sex-related differences in *B. caballi* seroprevalence were evident in the present study, with intact males exhibiting significantly higher seroprevalence than females and castrated males suggesting that sex- or management- related factors may influence exposure risk. The role of host sex and associated hormonal factors in susceptibility to *B. caballi* infection remains unclear [53]. Epidemiological evidence from different regions is variable, with some studies reporting higher infection rates in males [54], whereas others have found no significant association between seropositivity and gender [55].

No statistically significant associations were identified between breed or the number of horses per stable and seropositivity to any of the investigated pathogens, indicating that infection risk is unlikely to be driven by intrinsic host characteristics or stable size. Although differences between breeds have occasionally been reported, these are generally attributed to variations in management practices and environmental exposure rather than genetic susceptibility. Similarly, age was not significantly associated with seropositivity, whether analyzed as a continuous or a categorical variable. While some studies have suggested that older animals may exhibit higher infection rates, others have not identified a significant relationship between age and seroprevalence [38,56].

Housing type was found to be associated with *T. gondii* seropositivity at both the individual animal and stable level. The finding aligns with results of previous studies indicating that equids raised under extensive or free-ranging conditions show higher infection rates due to increased environmental exposure to oocysts shed by felids [57]. At the same time, other studies have reported elevated seroprevalence in more intensive or farm-based systems, mostly associated with contamination of feed and water sources within the stable environment [38].

In the present study, no significant associations were observed between seropositivity to *B. caballi* or *T. gondii* and management-related factors such as horse use, routine ectoparasiticide application, or prior illness history. These findings suggest that infection risk is more strongly influenced by environmental exposure than by animal use or recorded clinical history. The lack of a protective effect of ectoparasiticide use may reflect inconsistent application or variable efficacy, whereas the absence of association with prior illness reflects the subclinical nature of these infections.

Environmental variables, particularly precipitation and temperature-related predictors, were associated with predicted suitability patterns for *B. caballi*, consistent with previous studies on vector-borne infections in Mediterranean ecosystems [58,59,60]. In contrast, the ecological niche model developed for *T. gondii* showed poor predictive performance and high variability among cross-validation replicates, indicating limited capacity to reliably characterize environmental suitability patterns for this pathogen. The differences in performance between MaxEnt and Random Forest likely reflect differences in the underlying data structure, as MaxEnt relied on presence-only records, whereas Random Forest incorporated both presence and absence information. For *B. caballi*, precipitation of the driest month (BIO14) and minimum temperature of the coldest month (BIO6) emerged as the most influential environmental predictors. Given the poor performance and instability of the *T. gondii* model, no robust ecological interpretation can be drawn for this pathogen.

## 5. Conclusions

Tick-borne and zoonotic diseases are driven by complex interactions among hosts, vectors, environmental conditions, and anthropogenic changes in land use and climate. Equids are frequently involved in the transmission and maintenance cycles of pathogens of veterinary and public health relevance, often reflecting pathogen circulation within shared ecosystems.

This study should be interpreted in light of several limitations. First, the use of convenience sampling based on voluntary owner participation may have introduced selection bias, and therefore the study population may not be fully representative of the broader equid population in Southwestern Greece. Also, given the relatively limited number of animals sampled per stable, stable-level results should be interpreted as indicators of pathogen presence rather than precise estimates of within-stable prevalence. Second, no formal sample size calculation was performed before sampling, and the low number of seropositive animals for some pathogens limited statistical power. Third, inconclusive serological results were classified as negative after repeat testing, which may have underestimated true seroprevalence, although in any case the possible impact of this with 2/159 relevant samples would be rather small. Fourth, the cross-sectional design does not allow temporal or causal inference. Finally, the ecological niche models were based on limited positive observations and correlative environmental data; therefore, their outputs should be considered exploratory.

Furthermore, the multifactorial nature of pathogen exposure and the limited number of seropositive observations may complicate the interpretation of epidemiological associations, particularly because extensive adjustment for confounding factors and formal assessment of multicollinearity were not feasible. Consequently, the reported findings should be interpreted with appropriate caution.

Overall, the findings provide preliminary epidemiological information regarding exposure of equids in Southwestern Greece to selected vector-borne and zoonotic pathogens and contribute to a better understanding of their environmental and epidemiological context.

## Figures and Tables

**Table 1:** Bioclimatic and environmental variables included in the ecological niche modelling analysis.

Variable Code	Variable Name	Description
BΙO1	Annual mean temperature	Average annual temperature
BΙO2	Mean Diurnal Range	Mean monthly difference between maximum and minimum temperatures
BΙO3	Isothermality	Ratio of mean diurnal temperature range to annual temperature range (%)
BΙO4	Temperature Seasonality	Standard deviation of monthly temperatures × 100
BΙO5	Maximum temperature of warmest month	Highest temperature of the warmest month
BΙO6	Minimum temperature of coldest month	Lowest temperature of the coldest month
BΙO7	Temperature annual range	Difference between maximum and minimum temperature
BΙO8	Mean temperature of wettest quarter	Average temperature during the wettest quarter
BΙO9	Mean temperature of driest quarter	Average temperature during the driest quarter
BΙO10	Mean temperature of warmest quarter	Average temperature during the warmest quarter
BΙO11	Mean temperature of coldest quarter	Average temperature during the coldest quarter
BΙO12	Annual precipitation	Total annual precipitation
BΙO13	Precipitation of wettest month	Total precipitation of the wettest month
BΙO14	Precipitation of driest month	Total precipitation of the driest month
BΙO15	Precipitation Seasonality	Coefficient of variation in monthly precipitation
BΙO16	Precipitation of wettest quarter	Total precipitation during the wettest quarter
BΙO17	Precipitation of driest quarter	Total precipitation during the driest quarter
BΙO18	Precipitation of warmest quarter	Total precipitation during the warmest quarter
BΙO19	Precipitation of coldest quarter	Total precipitation during the coldest quarter
LandCorine	Land use/land cover	CORINE Land Cover 2018 classification describing land-use and land-cover categories within the study area
Elevation	Elevation	Altitude above sea level (m) extracted from the WorldClim digital elevation model

**Table 2:** Association between host-related factors (sex, breed, and age) and seropositivity to *B. caballi* and *T. gondii* in equids from Southwestern Greece. Data are presented as the total number of animals sampled (*N*), number of seropositive animals (*n*), and corresponding percentages (%).

		Seropositivity Against *B. caballi*				Seropositivity Against *T. gondii*			
	*N*	*n*	%	*χ* ^2^	*p*-Value	*n*	%	*χ* ^2^	*p*-Value
**Sex**				9.75	0.008			0.02	0.988
Intact Male	63	11	17.46			5	7.93		
Female	69	2	2.89			5	7.24		
Castrated Male	27	1	3.70			2	7.40		
**Breed**				-	>0.05			-	>0.05
Andalusian	11	0	0.00			0	0		
Friesian	9	1	11.11			1	11.11		
Mixed breed	81	8	9.87			8	9.87		
^1^ Penia	20	2	10.00			1	5.00		
Pinto	4	0	0.00			0	0.00		
Warmblood	14	1	7.14			1	7.14		
Warmblood cross	8	0	0.00			0	0.00		
**Age**				-	>0.05			-	>0.05
Young (<4 y)	6	0	0.00			1	16.67		
Young adult (4–10 y)	57	6	10.52			3	5.26		
Adult (11–14 y)	31	5	16.12			4	12.9		
Senior (>14 y)	65	3	4.61			4	6.53		

Note: ^1^ *Penia*, refers to a local Greek equine population not formally classified as a standardized breed; Ponies, donkeys and horses belonging to minor breeds were not included in breed-based comparisons.

**Table 3:** Association between stable-level management and environmental factors and seropositivity to *B. caballi* and *T. gondii* in equids from Southwestern Greece. Data are presented as the total number of stables within each category (*N*), number of seropositive stables (*n*), and corresponding percentages (%). A stable was considered positive when at least one sampled equid tested seropositive.

		Seropositivity Against *B. caballi*				Seropositivity Against *T. gondii*			
	*N*	*n*	%	*χ* ^2^	*p*-Value	*n*	%	*χ* ^2^	*p*-Value
**Housing Type**				3.45	0.18			**6.92**	**0.03**
Closed	20	2	10.00			2	10.00		
Semi-open	11	4	36.36			5	45.45		
Outdoor	24	7	29.17			3	12.50		
**Farm Animals**				2.76	0.10			3.26	0.07
Presence	36	11	30.56			9	25.00		
Absence	19	2	10.52			1	5.26		
**Companion** **Animals**				0.35	0.55			1.50	0.22
Presence	49	11	22.45			10	20.40		
Absence	6	2	33.33			0	0.00		
**Wildlife**				0.48	0.49			0.10	0.75
Presence	25	7	28.00			5	20.00		
Absence	30	6	20.00			5	16.67		
**Equid Use**				0.18	0.67			0.01	0.92
Working	6	1	16.67			1	16.67		
Leisure	49	12	24.49			9	18.37		
**Routine** **Ectoparasiticide Treatment**				1.06	0.30			0.58	0.45
Yes	28	5	17.86			4	14.29		
No	27	8	29.62			6	22.22		

**Table 4:** Predictive performance (AUC) and relative importance of environmental predictors in MaxEnt and Random Forest models for *B. caballi* and *T. gondii*. MaxEnt values represent average percentage contributions across cross-validation replicates, whereas Random Forest values represent variable-importance scores.

Pathogen	Model	AUC	BIO14	BIO6	Elevation	Land Cover
*B. caballi*	MaxEnt	0.705	64.3	35.7	0.00	0.00
	RF	0.504	16.2	26.4	40.5	16.9
*T. gondii* ^1^	MaxEnt	0.397	21.5	23.2	13.9	11.1
	RF	0.730	14.5	30.4	43.5	11.7

^1^ For *T. gondii*, variable-importance estimates should be interpreted cautiously, because of low predictive performance and high variability among cross-validation replicates.

## Data Availability

The original contributions presented in this study are included in the article. Further inquiries can be directed to the corresponding author.
